# Waiting for cholecystectomy: determinants of prioritization for delayed cholecystectomy in a universal public healthcare system—*post hoc* analysis of RELAPSTONE cohort (WAIT-CHOL study)

**DOI:** 10.1093/bjsopen/zrag021

**Published:** 2026-04-15

**Authors:** Raúl Velamazán, Daniel Oyón, Juan Lerma-Irureta, Pablo López-Guillén, Samuel J Martínez-Domínguez, Anna Arnau, Daniel Abad Baroja, Lara M Ruiz-Belmonte, Javier Tejedor-Tejada, Raul Zapater, Noelia Martín-Vicente, Pedro José Fernández-Esparcia, Ana Belén Julián Gomara, Violeta Sastre Lozano, Juan José Manzanares García, Irene Chivato Martín-Falquina, Laura Andrés Pascual, Nuria Torres Monclus, Natividad Zaragoza Velasco, Eukene Rojo, Pablo Cañamares-Orbís, Laura Pardo Grau, María Vaamonde Lorenzo, Arantzazu Izagirre Arostegi, Virginia Flores, Arantxa Diaz Gomez, Ana Garcia Garcia de Paredes, Berta Lapeña-Muñoz, Guillermo García-Rayado, Judith Millastre Bocos, Vicente Borrego, Carlos Sostres, Angel Lanas, Jose Manuel Ramia, Enrique de-Madaria

**Affiliations:** Department of Gastroenterology, Hospital Clínico Universitario Lozano Blesa, Zaragoza, Spain; IIS (Instituto de Investigacion Sanitaria) Aragón, Zaragoza, Spain; Centro de Investigacion Biomedica en Red de Enfermedades Hepaticas y Digestivas (CIBERehd) Instituto de Salud Carlos III, Madrid, Spain; Department of Gastroenterology, Hospital General Universitario Gregorio Marañón, Madrid, Spain; IIS (Instituto de Investigacion Sanitaria) Aragón, Zaragoza, Spain; Department of Gastroenterology, Hospital Universitario de Torrevieja, Alicante, Spain; Department of Gastroenterology, Hospital Clínico Universitario Lozano Blesa, Zaragoza, Spain; IIS (Instituto de Investigacion Sanitaria) Aragón, Zaragoza, Spain; Centro de Investigacion Biomedica en Red de Enfermedades Hepaticas y Digestivas (CIBERehd) Instituto de Salud Carlos III, Madrid, Spain; Research and Innovation Unit, Althaia Xarxa Assistencial Universitària de Manresa, Manresa, Spain; Central Catalonia Chronicity Research Group (C3RG), Centre for Health and Social Care Research (CESS), University of Vic-Central University of Catalonia (UVIC-UCC), Vic, Spain; Faculty of Medicine, University of Vic-Central University of Catalonia (UVIC-UCC), Vic, Spain; IIS (Instituto de Investigacion Sanitaria) Aragón, Zaragoza, Spain; Department of Gastroenterology, Hospital Universitario Miguel Servet, Zaragoza, Spain; Department of Gastroenterology, Hospital Clínico Universitario Lozano Blesa, Zaragoza, Spain; Department of Gastroenterology, Hospital Universitario Río Hortega, Valladolid, Spain; Department of Gastroenterology and Hepatology, Hospital Universitario Ramón y Cajal, Madrid, Spain; Department of Gastroenterology, Hospital de Galdakao, Bizkaia, Spain; Miguel Hernandez University, Elche, Spain; Department of Gastroenterology, Hospital Universitario Miguel Servet, Zaragoza, Spain; Department of Gastroenterology, Hospital Universitario Santa Lucia, Cartagena, Spain; Department of Gastroenterology, Hospital Universitario Santa Lucia, Cartagena, Spain; Department of Gastroenterology, Hospital Universitario de Burgos, Burgos, Spain; Department of Gastroenterology, Hospital Universitario de Burgos, Burgos, Spain; Department of Gastroenterology, Hospital Universitario Arnau de Vilanova, Lleida, Spain; Department of Gastroenterology, Hospital Universitario Arnau de Vilanova, Lleida, Spain; Department of Gastroenterology, Hospital Universitario de La Princesa, Madrid, Spain; IIS (Instituto de Investigación Sanitaria)-Princesa, Madrid, Spain; Department of Gastroenterology, Hospital de Galdakao, Bizkaia, Spain; Department of Gastroenterology, Hospital Universitario Josep Trueta, Girona, Spain; Department of Gastroenterology, Hospital Universitario Donostia, Donostia, Spain; Department of Gastroenterology, Hospital Universitario Donostia, Donostia, Spain; Department of Gastroenterology, Hospital General Universitario Gregorio Marañón, Madrid, Spain; Department of Gastroenterology, Hospital General Universitario Gregorio Marañón, Madrid, Spain; Centro de Investigacion Biomedica en Red de Enfermedades Hepaticas y Digestivas (CIBERehd) Instituto de Salud Carlos III, Madrid, Spain; Department of Gastroenterology and Hepatology, Hospital Universitario Ramón y Cajal, Madrid, Spain; Universidad de Alcalá, Madrid, Spain; IRYCIS (Instituto Ramón y Cajal de Investigación Sanitaria), Madrid, Spain; Department of Gastroenterology, Hospital Universitario San Pedro, Logroño, Spain; Department of Gastroenterology, Hospital Clínico Universitario Lozano Blesa, Zaragoza, Spain; IIS (Instituto de Investigacion Sanitaria) Aragón, Zaragoza, Spain; Department of Gastroenterology, Hospital Clínico Universitario Lozano Blesa, Zaragoza, Spain; IIS (Instituto de Investigacion Sanitaria) Aragón, Zaragoza, Spain; Department of Surgery, Hospital Clínico Universitario Lozano Blesa, Zaragoza, Spain; Department of Gastroenterology, Hospital Clínico Universitario Lozano Blesa, Zaragoza, Spain; IIS (Instituto de Investigacion Sanitaria) Aragón, Zaragoza, Spain; Centro de Investigacion Biomedica en Red de Enfermedades Hepaticas y Digestivas (CIBERehd) Instituto de Salud Carlos III, Madrid, Spain; Department of Gastroenterology, Hospital Clínico Universitario Lozano Blesa, Zaragoza, Spain; IIS (Instituto de Investigacion Sanitaria) Aragón, Zaragoza, Spain; Centro de Investigacion Biomedica en Red de Enfermedades Hepaticas y Digestivas (CIBERehd) Instituto de Salud Carlos III, Madrid, Spain; Department of Surgery, Hospital General Universitario Dr Balmis, Alicante, Spain; Department of Gastroenterology, Hospital General Universitario Dr Balmis-ISABIAL, Alicante, Spain; Department of Clinical Medicine, Miguel Hernandez University, Elche, Spain

**Keywords:** acute cholecystitis, gallstone disease, waiting list time

## Abstract

**Background:**

Symptomatic gallstone disease is a common and burdensome condition, with early cholecystectomy recommended to prevent relapses. However, delayed cholecystectomies lack standardized prioritization criteria. This study aimed to identify determinants independently associated with shorter waiting times for delayed cholecystectomy, and to assess the alignment of current surgical prioritization with relapse predictors described in the previous RELAPSTONE study.

**Methods:**

This was a *post hoc* analysis of the Spanish RELAPSTONE cohort, comprising patients admitted for a first episode of symptomatic gallstone disease between January 2018 and April 2020, who did not undergo cholecystectomy during the index hospital admission. The primary outcome was waiting time (in months) to delayed cholecystectomy. Linear regression models with β-coefficients were used to identify clinical factors associated with surgical delays (primary outcome of interest). Secondarily, the impact of relapse on prioritization was also evaluated.

**Results:**

This study analysed 1508 patients of the 3016 included in the RELAPSTONE cohort. Median age was 68.2 (interquartile range 53.9–76.6) years; 51.4% were men. Median waiting time to delayed cholecystectomy was 4.5 (interquartile range 2.3–7.0) months. Initial presentation as acute cholecystitis (β = −2.2, *P* = 0.048) and multiple cholelithiasis (β = −0.6, *P* = 0.006) were linked to shorter waiting times, whereas older age (> 54 years; β = 0.8, *P* = 0.002), advanced liver disease (β = 2.4, *P* = 0.047), and relapse (β = 0.5, *P* = 0.036) were associated with longer delays, compared with their respective reference groups. Among 575 patients (38.1%) with relapse, time to delayed cholecystectomy did not differ by relapse type. Several known risk factors for relapse identified in previous RELAPSTONE analyses, including the performance of endoscopic sphincterotomy and leucocyte and alanine aminotransferase levels, did not influence prioritization.

**Conclusion:**

Determinants of surgical timing were not well aligned with previously identified predictors of relapse. These findings support the need for evidence-based triage strategies to improve timing and outcomes of delayed cholecystectomy.

## Teaser

Using data from 1508 patients across 16 Spanish hospitals, factors influencing surgical waiting times for delayed cholecystectomy were investigated. Current prioritization was found to be largely empirical and misaligned with risk of relapse. Evidence-based triage models are urgently needed to protect high-risk patients.

## Introduction

Gallstones are a highly prevalent condition, and more than one in five individuals develop symptomatic gallstone disease (SGD) within 10 years of diagnosis^1,2^. SGD includes biliary colic (BC) and gallstone-related complications, such as acute biliary pancreatitis (AP), acute calculous cholecystitis (ACC), symptomatic choledocholithiasis (SC), and acute cholangitis (ACL), representing a major public health concern owing to its contribution to emergency department visits, hospital admissions, and surgical workload worldwide^3^. Early cholecystectomy is recommended universally to prevent recurrent biliary events, with timing of surgery being the strongest modifiable predictor of relapse^4–9^.

In Spain, early cholecystectomy is seldom performed during the index hospital admission because of limited surgical capacity. Consequently, most patients undergo delayed cholecystectomy (DC) after discharge. Despite multiple policy measures implemented over the past two decades, waiting times in Spain remain prolonged, currently averaging 119 days^10^. This could not only increase patient morbidity owing to the risk of relapse but also contribute to greater surgical complexity^11–13^.

In addition to timing of surgery, other clinical and demographic variables have been identified as potential risk factors for relapse, underscoring the need for a more refined approach to patient stratification. In a previous paper^14^, the authors’ group identified older age (> 54 years; hazard ratio (HR) 0.57, 95% confidence interval (c.i.) 0.49 to 0.66), sphincterotomy (HR 0.58, 0.49 to 0.68), and leucocytosis during admission (> 11 000/mm^3^; HR 0.79, 0.70 to 0.90) as prognostic determinants independently associated with a lower risk of relapse. In contrast, multiple cholelithiasis (HR 1.19, 1.05 to 1.34) and a raised level of alanine aminotransferase (ALT) during admission (> 35 units/l; HR 1.22, 1.02 to 1.46) were associated with an increased risk.

Despite this knowledge, no universally accepted prioritization framework for SGD exists. Current triage practices remain heterogeneous, and proposed prioritization criteria often rely on empirical considerations such as SGD subtype, co-morbidity, or relapse history^15–18^. Furthermore, most published models are derived from single-centre centres, lack robust validation, and have not shown effectiveness in reducing adverse outcomes^19,20^.

This evidence gap raises concerns about the equity and efficiency of current surgical prioritization. Notably, there is a striking lack of studies evaluating prioritization practices in high-burden settings with prolonged surgical delays, such as those in Spain.

The hypothesis was that current prioritization practices rely more on clinical practice rather than on evidence-based predictors of relapse, potentially leading to suboptimal allocation of surgical resources and inadequate protection of patients with high-risk disease.

In this context, the primary objective of this study was to identify determinants independently associated with shorter waiting times for DC. As secondary objectives, the impact of relapse was assessed, both its occurrence and in relation to SGD subtype, on surgical prioritization, and whether the determinants identified in the RELAPSTONE study were applicable in real-world prioritization practices in the authors’ cohort^14^.

## Methods

### Design

This study comprised a *post hoc* analysis of the RELAPSTONE cohort, an international multicentre retrospective study that included 16 tertiary Spanish hospitals and 2 Mexican hospitals^14^; a complete list of participating centres is available in the *supplementary materials*. Across centres, early cholecystectomy, defined as cholecystectomy performed during the index hospital admission, is recommended but implemented inconsistently, and DC, defined as surgery performed after hospital discharge,is therefore common in routine practice.

The study was approved by the central institutional review board (IRB) (Dr Balmis General University Hospital, reference 2020-257, 18 January 2021) and by the local IRBs of collaborating centres. Given the retrospective nature of the study and its independence from commercial interests, the IRBs waived the requirement for informed consent in accordance with Spanish law.

### Patient and data selection

The cohort comprised consecutive patients admitted for a first episode of SGD who did not undergo cholecystectomy during the index hospital admission, between 1 January 2018 and 30 April 2020. SGD was defined as the presence of: AP, according to the revised Atlanta classification^21^; ACC or ACL, according to the Tokyo guidelines 2018^22,23^; SC; BC; or any combination of these diagnoses. Patients were excluded if they died during the index admission, had a history of pancreatobiliary surgery or endoscopic sphincterotomy, had a diagnosis of biliary, duodenal, or pancreatic malignancy, or had benign biliary strictures.

All patients were followed until they underwent DC. DC was defined as any cholecystectomy performed after discharge from the index hospital admission, regardless of whether it was scheduled or carried out urgently when indicated clinically (for example in the event of relapse as ACC). Each participating hospital applied its own local prioritization protocols for surgical scheduling. Additionally, recurrent biliary events before surgery were documented, whether they required hospital admission or only emergency department evaluation.

A comprehensive set of variables potentially influencing surgical prioritization was analysed, including: demographic data; clinical characteristics; laboratory parameters at three different time points during hospital admission (at admission (first 24 h), at discharge (in the last 48 h of the admission), and the highest value of the parameter during admission); imaging findings; and the occurrence of relapses. All variables collected, along with their definitions, are provided in the *Supplementary material*. Data collection and patient follow-up were conducted by means of a systematic review of electronic health records at each participating centre. To ensure data reliability, all records were reviewed by experienced clinicians at each site, following a standardized data collection protocol.

This cohort study adhered to the STROBE guidelines^24^. All data were collected and analysed in anonymized form using a centralized database.

### Outcome of interest

The primary outcome was the waiting time to DC, defined as the number of months between discharge after the index hospital admission and the date of surgery. Secondary outcomes included the impact of relapse on surgical prioritization, assessed as the occurrence of relapse and its SGD subtype, and whether determinants identified in the RELAPSTONE study applied in real-world prioritization practices in the present cohort.

### Statistical analysis

Categorical variables are summarized as absolute numbers and percentages. Continuous variables are summarized as median (interquartile range, i.q.r.), given their non-normal distribution.

Simple and multiple linear regression models were used to identify determinants of waiting time for DC. The unadjusted and adjusted β-regression coefficients, along with 95% confidence intervals, were calculated to estimate effect size. For independent variables with less than 5% missing data, imputation was performed using the median for continuous variables and the mode for categorical variables.

Two multivariable models were developed. Model 1 included co-variables that met both of the following criteria: *P* ≤ 0.200 in the univariable analysis, and clinical relevance or previously established associations in the literature. Model 2 included only variables previously identified in the RELAPSTONE study as being associated with a higher risk of relapse. Statistical significance was set at a two-sided 5% level (*P* < 0.005). All *P* values were obtained from linear regression analyses. *P* values labelled as ‘model *P*’ correspond to the overall model significance, whereas the remaining *P* values correspond to the significance of individual regression coefficients (*t*-test). All analyses were conducted using R version 4.4.2 (R Core Team, Vienna, Austria), via the RStudio interface, and the heatmap was generated using the ‘pheatmap’ R package.

## Results

### Baseline patient characteristics and waiting times to cholecystectomy

Of 3016 patients included in the RELAPSTONE study, 1572 underwent DC. From this group, 52 patients from Mexican centres were excluded in order to focus exclusively on patients from tertiary Spanish hospitals, and 12 patients with surgery dates missing. The final study population comprised 1508 patients (*Fig. S1*).

The median age of the cohort was 68.2 (i.q.r. 53.9–76.6) years and 775 patients (51.4%) were men. Regarding co-morbidities, the majority of patients had low co-morbidity according to the Charlson Co-morbidity Index: 1101 (73.0%) scored 0–1, whereas 216 (14.3%) had moderate co-morbidity (2), and 191 (12.7%) had high co-morbidity (score ≥ 3). In terms of initial SGD, the most frequent condition was AP (564, 37.4%), followed by ACC (346, 22.9%). Of the 1508 DCs, 1407 (93.7%) were scheduled, and 101 (6.3%) were performed urgently. The main reasons for urgent cholecystectomy were relapse as ACC in 63 patients (62.4%) and as BC in 14 (13.9%).

The overall median waiting time to DC was 4.5 (i.q.r. 2.3–7.0) months, with a fairly even distribution across predefined time intervals: < 3 months for 510 patients (33.8%), 3–6 months for 477 (31.6%), and > 6 months for 521 (34.6%).

Demographic, clinical, and laboratory characteristics of the main clinically relevant variables, together with their associated median waiting times to DC and distribution across time intervals, are presented in *Table 1*. Additional variables are detailed in *Tables S1* and *S2*. The median waiting time to cholecystectomy across participating hospitals is shown in *Table S3*; there was substantial interhospital variation, with median waiting times ranging from 2.3 to 7.2 months.

**Table 1:** Baseline characteristics, median waiting time to cholecystectomy, and distribution of cholecystectomies by waiting time in relation to demographic and clinical variables

	*n* patients†	*n*	Waiting time to cholecystectomy (months)*	Waiting time (months)		
				< 3	3–6	> 6
All patients	1508	1508 (100%)	4.5 (2.3–7.0)	510 (33.8%)	477 (31.6%)	521 (34.6%)
**Type of gallstone disease**	1508					
Acute pancreatitis		564 (37.4%)	4.5 (2.5–6.9)	187 (33.2%)	186 (33.0%)	191 (33.9%)
Acute cholecystitis		346 (22.9%)	4.4 (2.3–6.9)	120 (34.7%)	109 (31.5%)	117 (33.8%)
Acute cholangitis		146 (9.7%)	5.3 (3.2–7.4)	35 (24.0%)	51 (34.9%)	60 (41.1%)
Symptomatic choledocholithiasis		160 (10.6%)	4.1 (1.7–7.0)	65 (40.6%)	37 (23.1%)	58 (36.3%)
Biliary colic		176 (11.7%)	3.9 (2.2–6.2)	64 (36.4%)	64 (36.4%)	48 (27.3%)
Multiple diseases		116 (7.7%)	5.0 (2.2–8.3)	38 (32.8%)	30 (25.9%)	48 (41.4%)
**Acute pancreatitis‡**	564					
Mild		445 (78.9%)	4.3 (2.3–6.7)	158 (35.5%)	151 (33.9%)	136 (30.6%)
Moderate		93 (16.5%)	5.0 (2.9–7.1)	25 (26.9%)	31 (33.3%)	37 (39.8%)
Severe		26 (4.6%)	7.3 (4.8–13.2)	4 (15.4%)	4 (15.4%)	18 (69.2%)
**Acute cholecystitis‡**	346					
Mild		262 (75.7%)	4.6 (2.5–7.0)	84 (32.1%)	87 (33.2%)	91 (34.7%)
Moderate		72 (20.8%)	3.7 (2.0–6.3)	30 (41.7%)	20 (27.8%)	22 (30.6%)
Severe		12 (3.5%)	3.3 (2.0–6.9)	6 (50.0%)	2 (16.7%)	4 (33.3%)
**Acute cholangitis‡**	146					
Mild		91 (62.3%)	4.1 (2.5–6.8)	27 (29.7%)	36 (39.6%)	28 (30.8%)
Moderate		45 (30.8%)	6.9 (5.4–9.2)	6 (13.3%)	11 (24.4%)	28 (62.2%)
Severe		10 (6.8%)	5.3 (3.8–8.0)	2 (20.0%)	4 (40.0%)	4 (40.0%)
**Age (years)**	1508					
≤ 54		380 (25.2%)	3.7 (1.9–6.4)	167 (43.9%)	101 (26.6%)	112 (29.5%)
> 54		1128 (74.8%)	4.6 (2.5–7.1)	342 (30.3%)	376 (33.3%)	410 (36.3%)
**Sex**	1508					
Female		733 (48.6%)	4.2 (2.2–6.7)	260 (35.5%)	235 (32.1%)	238 (32.5%)
Male		775 (51.4%)	4.7 (2.5–7.2)	249 (32.1%)	242 (31.2%)	284 (36.6%)
**Charlson Co-morbidity Index score**	1508					
0–1 (low co-morbidity)		1101 (73.0%)	4.3 (2.3–6.9)	385 (35.0%)	349 (31.7%)	367 (33.3%)
2 (medium co-morbidity)		216 (14.3%)	5.1 (2.8–7.3)	60 (27.8%)	74 (34.3%)	82 (38.0%)
≥ 3 (high co-morbidity)		191 (12.7%)	4.6 (2.2–7.1)	64 (33.5%)	54 (28.3%)	73 (38.2%)
**Liver disease**	1508					
None		1447 (95.7%)	4.5 (2.3–6.9)	485 (33.5%)	464 (32.1%)	498 (34.4%)
Mild		47 (3.1%)	3.6 (2.3–8.0)	19 (40.4%)	11 (23.4%)	17 (36.2%)
Moderate or severe		14 (0.9%)	6.1 (2.4–8.3)	5 (35.7%)	2 (14.3%)	7 (50.0%)
**Multiple cholelithiasis**	1508					
No		677 (44.9%)	4.8 (2.3–7.2)	220 (32.5%)	204 (30.1%)	253 (37.4%)
Yes		831 (55.1%)	4.1 (2.3–6.9)	289 (34.8%)	273 (32.9%)	269 (32.4%)
**Acute necrotic collection or walled-off necrosis**	1508					
No		1447 (95.9%)	4.4 (2.3–6.9)	499 (34.5%)	455 (31.4%)	493 (34.1%)
Yes		61 (4.1%)	5.9 (3.9–8.7)	10 (16.4%)	22 (36.1%)	29 (47.5%)
**Sphincterotomy**	1508					
No		1158 (76.8%)	4.5 (2.4–7.0)	380 (32.8%)	374 (32.3%)	404 (34.9%)
Yes		350 (23.2%)	4.3 (2.2–6.9)	129 (36.9%)	103 (29.4%)	118 (33.7%)
**Highest ALT value (units/l)**	1508					
≤ 35		209 (13.8%)	4.6 (2.2–7.2)	73 (34.9%)	65 (31.1%)	71 (34.0%)
> 35		1299 (86.0%)	4.4 (2.3–6.9)	436 (33.6%)	412 (31.7%)	451 (34.7%)
**Highest leucocyte value (counts/mm^3^)**	1508					
≤ 11 000		687 (45.5%)	4.1 (2.2–6.5)	246 (35.8%)	234 (34.1%)	207 (30.1%)
> 11 000		821 (54.3%)	4.9 (2.4–7.3)	263 (32.0%)	243 (29.6%)	315 (38.4%)
**Relapse**	1508					
No		933 (61.9%)	4.4 (2.4–6.8)	309 (33.1%)	308 (33.0%)	316 (33.9%)
Yes		575 (38.1%)	4.5 (2.2–7.5)	200 (34.8%)	169 (29.4%)	206 (35.8%)

Values are *n* (%) unless otherwise stated; *values are median (interquartile range). †Number of available observations for each variable. ‡Severity was graded according to the Atlanta classification (2012) for acute pancreatitis, and the Tokyo classification (2018) for acute cholecystitis and cholangitis. ALT, alanine aminotransferase.

### Determinants of waiting times for cholecystectomy

In the bivariable analysis, shorter waiting times to DC were observed for patients with ACC and BC, milder initial presentations of AP and ACL, younger age, lower Charlson Co-morbidity Index scores, absence of advanced liver disease, presence of multiple cholelithiasis, absence of evidence of acute necrotic collections or walled-off necrosis, and no history of relapse (*Table 2*). Additional variables and their associated median waiting times to DC are detailed in *Tables S1* and *S2*; significant differences in waiting time were observed for selected clinical and laboratory factors, including previous alcohol use, acute respiratory dysfunction, positive bile cultures, percutaneous cholecystostomy, leucocyte count and C-reactive protein level at admission, haematocrit at discharge, and highest urea value.

**Table 2:** Clinical and demographic factors associated with waiting time to cholecystectomy: unadjusted coefficients according to bivariable linear regression model

	Waiting time to cholecystectomy (months)*	Bivariable analysis		
		β-coefficient†	*P*	Model *P*
**Type of gallstone disease**				0.053
Acute pancreatitis	4.5 (2.5–6.9)	−0.7 (−1.5, 0.1)	0.099	
Acute cholecystitis	4.4 (2.3–6.9)	−0.9 (−1.8, −0.1)	0.039	
Acute cholangitis	5.3 (3.2–7.4)	0 (reference)		
Symptomatic choledocholithiasis	4.1 (1.7–7.0)	−0.7 (−1.7, 0.3)	0.167	
Biliary colic	3.9 (2.2–6.2)	−1.5 (−2.5, −0.5)	0.002	
Multiple diseases	5.0 (2.2–8.3)	−0.3 (−1.4, 0.8)	0.549	
**Acute pancreatitis**				0.001
Mild	4.3 (2.3–6.6)	0 (reference)		
Moderate	5.0 (2.9–7.1)	0.4 (−0.6, 1.4)	0.412	
Severe	7.3 (4.8–13.2)	3.4 (1.7, 5.2)	< 0.001	
**Acute cholecystitis**				0.189
Mild	4.6 (2.5–7.0)	0 (reference)		
Moderate	3.7 (2.0–6.3)	−1.0 (−2.1, 0.1)	0.076	
Severe	3.3 (2.0–6.9)	−0.8 (−3.2, 1.7)	0.545	
**Acute cholangitis**				< 0.001
Mild	4.1 (2.5–6.8)	0 (reference)		
Moderate	6.9 (5.4–9.2)	3.8 (2.1, 5.5)	< 0.001	
Severe	5.3 (3.8–8.0)	1.3 (−1.9, 4.4)	0.432	
**Age (years)**				< 0.001
≤ 54	3.7 (1.9–6.4)	0 (reference)		
> 54	4.6 (2.5–7.1)	1.0 (0.5, 1.5)		
**Sex**				0.100
Female	4.2 (2.2–6.7)	0 (reference)		
Male	4.7 (2.5–7.2)	0.4 (−0.1, 0.8)		
**Charlson Co-morbidity Index score**				0.014
0–1 (low co-morbidity)	4.3 (2.3–6.9)	0 (reference)		
2 (medium co-morbidity)	5.1 (2.8–7.3)	1.0 (0.3, 1.6)	0.004	
3 (high co-morbidity)	4.6 (2.2–7.1)	0.3 (−0.4, 1.0)	0.357	
**Liver disease**				0.044
None	4.5 (2.3–6.9)	0 (reference)		
Mild	3.6 (2.3–8.0)	0.9 (−0.4, 2.2)	0.163	
Moderate or severe	6.1 (2.4–8.3)	2.5 (0.2, 4.9)	0.035	
**Multiple cholelithiasis**				0.006
No	4.8 (2.3–7.2)	0 (reference)		
Yes	4.1 (2.3–6.9)	−0.6 (−1.1, −0.2)		
**Acute necrotic collection or walled-off necrosis**				0.007
No	4.4 (2.3–6.9)	0 (reference)		
Yes	5.9 (3.9–8.7)	1.6 (0.4, 2.7)		
**Sphincterotomy**				0.921
No	4.5 (2.4–7.0)	0 (reference)		
Yes	4.3 (2.2–6.9)	0.1 (−0.6, −0.5)		
**Highest ALT value (units/l)**				0.932
≤ 35	4.6 (2.2–7.2)	0 (reference)		
> 35	4.4 (2.3–6.9)	0.1 (−0.6, 0.7)		
**Highest leucocyte value (counts/mm^3^)**				0.057
≤ 11 000	4.1 (2.2–6.5)	0 (reference)		
> 11 000	4.9 (2.4–7.3)	0.4 (−0.0, 0.9)		
**Relapse**				0.027
No	4.4 (2.4–6.8)	0 (reference)		
Yes	4.5 (2.2–7.5)	0.5 (0.1, 1.0)		

*Values are median (interquartile range); †values in parentheses are 95% confidence intervals. *P* (*t*-test for regression coefficients). ALT, alanine aminotransferase.

In the multivariable analysis, the determinants independently associated with shorter waiting times were the initial presentation of SGD as ACC (β = −2.2, 95% c.i. −4.3 to 0.0; *P* = 0.048) and the presence of multiple cholelithiasis (β = −0.6, −1.1 to −0.2; *P* = 0.006). Older age (> 54 years) (β = 0.8, 0.3 to 1.4; *P* = 0.002), presence of advanced liver disease (β = 2.4, 0.0 to 4.7; *P* = 0.047), and having experienced at least one relapse during follow-up (β = 0.5, 0.0 to 1.0; *P* = 0.036) were independently associated with longer delays to surgery (model 1, *Table 3*).

**Table 3:** Clinical and demographic factors associated with waiting time to cholecystectomy: adjusted coefficients according to multivariable linear regression models

	Multivariable analysis			
	Model 1* (model *P* < 0.001)		Model 2† (model *P* < 0.001)	
	β-coefficient	*P*	β-coefficient	*P*
**Type of gallstone disease**			–	
Acute pancreatitis	−0.8 (−3.4, 1.7)	0.532		
Acute cholecystitis	−2.2 (−4.3, 0.0)	0.048		
Acute cholangitis	0 (reference)			
Symptomatic choledocholithiasis	−2.3 (−6.5, 1.9)	0.288		
Biliary colic	−3.0 (−7.3, 1.2)	0.156		
Multiple diseases	0.1 (−4.7, 5.0)	0.956		
**Acute pancreatitis**			–	
Mild	−1.7 (−6.2, 2.8)	0.451		
Moderate	−1.4 (−6.0, 3.2)	0.555		
Severe	1.2 (−3.7, 6.0)	0.635		
**Acute cholecystitis**			–	
Mild	−0.4 (−4.6, 3.9)	0.873		
Moderate	−1.0 (−5.2, 3.3)	0.665		
Severe	−0.8 (−5.6, 4.1)	0.754		
**Acute cholangitis**			–	
Mild	−3.0 (−7.1, 1.2)	0.166		
Moderate	0.6 (−3.5, 4.6)	0.791		
Severe	−2.9 (−7.7, 1.8)	0.221		
**Age (years)**				
≤ 54	0 (reference)		0 (reference)	
> 54	0.8 (0.3, 1.4)	0.002	1.0 (0.4, 1.5)	< 0.001
**Liver disease**			–	
None	0 (reference)			
Mild	0.8 (−0.5, 2.1)	0.244		
Moderate or severe	2.4 (0.0, 4.7)	0.047		
**Multiple cholelithiasis**				
No	0 (reference)		0 (reference)	
Yes	−0.6 (−1.1, −0.2)	0.006	−0.6 (−1.0, −0.1)	0.012
**Acute necrotic collection or walled-off necrosis**			–	
No	0 (reference)			
Yes	0.6 (−0.9, 2.0)	0.447		
**Sphincterotomy**	–			
No			0 (reference)	
Yes			−0.1 (−0.6, 0.5)	0.969
**Highest ALT value (units/l)**	–			
≤ 35			0 (reference)	
> 35			0.2 (−0.5, 0.8)	0.625
**Highest leucocyte value (counts/mm^3^)**	–			
≤ 11 000			0 (reference)	
> 11 000			0.4 (−0.1, 0.8)	0.111
**Relapse**			–	
No	0 (reference)			
Yes	0.5 (0.0, 1.0)	0.036		

Values in parentheses are 95% confidence intervals. *Model 1 included co-variables that met both of the following criteria: *P* ≤ 0.200 in univariable analysis, and clinical relevance or associations previously established in the literature. †Model 2 included only variables identified previously in the RELAPSTONE study as being associated with a higher risk of relapse. *P* (*t*-test for regression coefficients). −, Variable not introduced into the model; ALT, alanine aminotransferase.

Other variables previously identified in the RELAPSTONE study as predictors of recurrence, such as previous sphincterotomy, raised ALT levels, or leucocytosis during admission, were not significantly associated with waiting time to DC (model 2, *Table 3*).

**Fig. 1:**
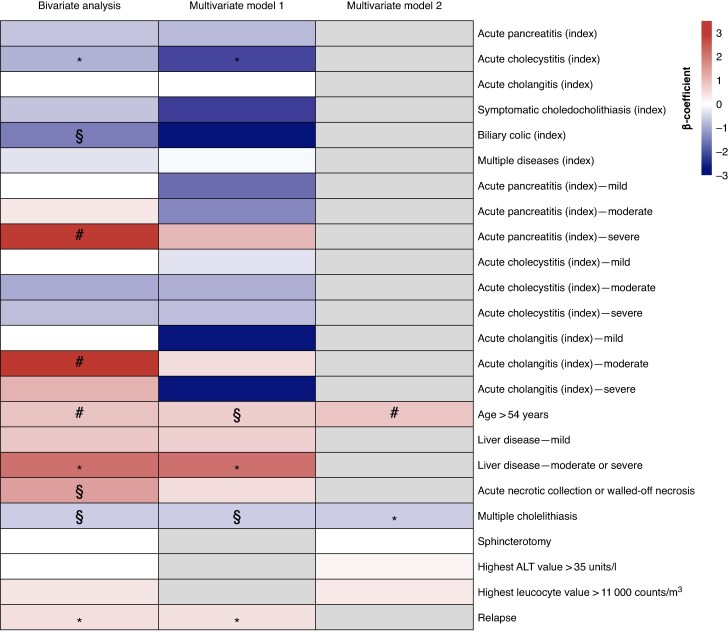
Heatmap of variables included in bivariable and multivariable analyses *P < 0.050, §*P* < 0.005, #*P* < 0.001. ALT, alanine aminotransferase.

*Figure 1* shows a heatmap of variables included in the bivariable and multivariable analyses, based on their association with waiting time to DC, with their statistical significance.

### Impact of relapse on surgical prioritization

A total of 575 patients (38.1%) experienced at least one relapse before undergoing DC. The median time from hospital discharge to relapse was 1.6 (i.q.r. 0.5–3.7) months and the median time from relapse to DC was 1.9 (0.6–4.3) months. Overall, the median time from hospital discharge to DC among patients who experienced a relapse was 4.5 (2.2–7.5) months, compared with 4.4 (2.4–6.8) months for those without relapse (*P* = 0.027). The distribution of relapse rates, waiting times until relapse, and waiting times to cholecystectomy, by type of SGD at initial presentation, is shown in *Table 4*.

**Table 4:** Influence of relapse on waiting time by type of gallstone disease at admission and in the overall sample

Reason for index admission	*n* patients	Relapse before cholecystectomy	*n*	Time to relapse (months)*	Time from relapse to cholecystectomy (months)*	Waiting time to cholecystectomy (months)*
Acute pancreatitis	564	No	352 (62.4%)			4.5 (2.4–6.8)
		Yes	212 (37.6%)	1.8 (0.6–3.4)	1.8 (0.6–4.5)	4.5 (2.5–7.2)
Acute cholecystitis	346	No	215 (62.3%)			4.4 (2.5–6.8)
		Yes	131 (37.8%)	1.8 (0.6–4.0)	1.7 (0.3− 3.6)	4.4 (2.0–7.0)
Acute cholangitis	146	No	84 (57.5%)			4.9 (2.7–6.9)
		Yes	62 (42.5%)	1.7 (0.5–4.8)	2.8 (0.6–5.5)	5.8 (3.7–8.9)
Symptomatic choledocholithiasis	160	No	107 (66.9%)			4.4 (1.6–6.7)
		Yes	53 (33.1%)	1.0 (0.4–4.0)	1.8 (0.7–3.8)	3.8 (1.9–7.6)
Biliary colic	176	No	99 (56.3%)			4.0 (2.5–6.2)
		Yes	77 (43.8%)	1.4 (0.4–3.3)	1.6 (0.5–3.1)	3.7 (1.5–6.1)
Multiple diseases	116	No	76 (65.5%)			5.0 (2.2–7.7)
		Yes	40 (34.5%)	1.3 (0.4–4.9)	2.1 (0.7–4.9)	4.9 (2.6–8.5)
All patients	1508	No	933 (61.9%)			4.4 (2.4–6.8)‡
		Yes	575 (38.1%)	1.6 (0.5–3.7)	1.9 (0.6–4.3)	4.5 (2.2–7.5)

Values are *n* (%) unless otherwise stated; *values are median (interquartile range). †Number of available observations for each variable. ‡*P =* 0.027 (*t*-test for regression coefficients).

No statistically significant differences were observed in waiting times according to type of SGD at the time of relapse (*P* = 0.932). However, patients who relapsed with ACC had the shortest median waiting time to surgery among all relapse types, with a median of 3.6 (1.6–7.0) months. The distribution of waiting times from hospital discharge and from relapse to cholecystectomy, stratified by the type of SGD at recurrence, is shown in *Table 5*.

**Table 5:** Impact of type of relapse on waiting times and bivariable analysis

Type of relapse	*n* patients‡	Time from relapse to cholecystectomy (months)*	Waiting time to cholecystectomy (months)*	β-coefficient†	Unadjusted *P*	Unadjusted model *P*
All patients with relapse	575					
Acute pancreatitis	163	1.7 (0.7–4.5)	4.6 (2.4–7.5)	0 (reference)		0.932
Acute cholecystitis	117	0.4 (0.1–2.6)	3.6 (1.6–7.0)	−0.5 (−1.7, 0.8)	0.439	
Acute cholangitis	41	2.2 (0.9–5.3)	5.3 (2.8–8.7)	−0.1 (−1.9, 1.7)	0.924	
Symptomatic choledocholithiasis	38	1.8 (0.6–5.5)	4.4 (2.0–7.5)	−0.5 (−2.3, 1.3)	0.589	
Biliary colic	189	2.0 (0.9–3.5)	4.6 (2.7–7.5)	−0.4 (−1.5, 0.7)	0.430	
Multiple diseases	27	2.3 (0.9–4.9)	4.5 (3.1–9.8)	0.3 (−1.8, 2.4)	0.763	

*Values are median (interquartile range); †values in parentheses are 95% confidence intervals. ‡Number of available observations for each variable, *P* (*t*-test for regression coefficients).

## Discussion

The present study aimed to identify determinants independently associated with shorter waiting times for DC in a public healthcare system and to evaluate whether real-world prioritization practices align with known predictors of relapse. It was found that factors such as ACC at initial presentation and multiple cholelithiasis were associated with shorter waiting times, whereas older age, advanced liver disease, and previous relapse were linked to longer delays.

The median waiting time to DC in this study was 4.5 (i.q.r. 2.3–7.0) months, consistent with official data reported in Spain^10^. This delay is clearly excessive considering that current clinical guidelines recommend early cholecystectomy in most patients, except for specific situations such as AP with local complications^4–7^. Such prolonged delays likely contribute to increased patient morbidity and mortality. In the authors’ previous study^14^, the 3-month relapse rate was 21%, with a notably early median time to relapse of 2.1 (0.7–5.5) months. There is also strong evidence to suggest that DC increases both the cost per patient and the technical complexity of the procedure^12,25^.

Although guideline-recommended early cholecystectomy would effectively prevent most recurrent biliary events, its widespread implementation in Spain is currently limited by longstanding structural constraints^10^. The present findings reinforce the need for prioritization strategies on surgical waiting lists, ideally based on relapse risk. This multicentre study aimed to identify common determinants of prioritization in a country such as Spain, which is characterized by a universal public health system and chronically prolonged surgical waiting times.

In previous studies^15,16^, the type of SGD at admission has often been a key determinant of surgical priority, with BC typically receiving the lowest priority. However, in the present cohort, only patients with ACC had significantly shorter waiting times, and no statistically significant differences were found for other SGD types. The fact that BC did not have a longer waiting time than other SGDs may have been due to selection bias of patients with BC in this cohort, who had to be admitted to hospital because of their GSD. Therefore, these were more complicated cases of BC than the vast majority, which are generally managed on an outpatient basis and were not included in this study.

Older age (> 54 years) was independently associated with longer delays to DC. This is a controversial finding. Some studies^14,26,27^, including RELAPSTONE, have shown that younger patients are at higher risk of relapse, and others^17^ also observed longer delays in patients aged > 50 years in a Canadian setting. In contrast, other authors^15^ have proposed that patients aged > 65 years should be prioritized, probably because of their increased surgical risk and frailty, even if their risk of relapse is lower.

There is limited evidence regarding the influence of co-morbidities. Only one study^16^ has suggested prioritizing patients with diabetes or immunosuppression, more owing to the potential perioperative risk than an established association with relapse. In the present analysis, patients with moderate-to-severe liver disease experienced significantly longer waiting times, although none of the other co-morbidities were independently associated with surgical timing.

Patients with multiple cholelithiasis had significantly shorter waiting times than those with single stones or biliary sludge. This aligns with previous evidence indicating that biliary sludge may be managed more conservatively because of its potential for spontaneous resolution and lower relapse risk^15,28,29^.

Regarding the severity of the index episode, neither its classification nor the presence of pancreatic fluid collections was associated with surgical timing, probably influenced by the long surgical waiting time.

Interestingly, patients with relapse had slightly longer overall waiting times than those without. This finding contrasts with previous literature^15,16^, in which relapse was generally regarded as an indication for surgical prioritization. Similarly, another analysis of an Australian cohort^20^ also reported longer surgical delays in patients who had experienced relapses (126 *versus* 203 days). Possible explanations include interhospital variation in prioritization protocols, inability to reduce waiting times despite recognition of relapse, or required recovery periods following recurrent episodes that may delay scheduling. The fact that relapse was not a criterion for prioritization in this study is particularly concerning—not only because of its impact on patients’ quality of life but also because it may expose them to a higher risk of conversion to open surgery and iatrogenic bile duct injury during DC^12,25^.

It was also assessed whether the type of recurrent event influenced surgical timing. However, there was no significant difference across categories, although there was a trend, albeit not statistically significant, towards shorter waiting times for relapse in the form of ACC, probably explained by the large number of urgent cholecystectomies performed in this context (63 of 117).

Finally, it was evaluated whether real-world prioritization practices were aligned with recurrence risk determinants identified in the RELAPSTONE cohort^14^. Partial concordance was observed: older age (> 54 years), a protective factor against relapse, was associated with longer delays. On the other hand, the presence of multiple cholelithiasis, a risk factor for recurrence, was associated with shorter waiting times.

Other protective determinants, most notably previous endoscopic sphincterotomy, had no impact on surgical prioritization, despite strong supporting evidence from RELAPSTONE and other cohorts^30,31^. Similarly, leucocyte count and ALT levels during admission, which were associated with recurrence risk, were not associated with surgical timing.

These discrepancies highlight a persistent gap between evidence-based risk stratification and real-world prioritization decisions. This misalignment is likely driven by systemic constraints and variability in local institutional policies rather than by clinical reasoning alone.

Based on these results, an evidence-informed framework for surgical prioritization, adapted to each hospital’s structure and resources, should combine two elements: the surgeon’s assessment of operative risk and the estimated probability of disease recurrence.

Regarding recurrence risk, all SGDs other than BC should receive higher prioritization. Younger patients, those with multiple gallstones, and those presenting with additional but lower-impact predictors, such as raised ALT level during admission or absence of leucocytosis, may also warrant shorter waiting times. In contrast, patients who have undergone endoscopic sphincterotomy may be scheduled later, given its observed protective effect. If relapse occurs despite preventive prioritization, this event should contribute to prioritization decisions given its potential to complicate surgery. This proposal represents an initial conceptual framework; prospective validation studies are required before implementation into standardized prioritization algorithms.

The present study has several limitations. Its retrospective design inherently limits causal inference. Additionally, waiting time was defined as the interval from hospital discharge to DC, excluding intervening dates such as surgical consultation. However, given the organizational structure of the Spanish healthcare system, delays are more likely attributable to surgical scheduling rather than referral or consultation delays. Furthermore, it was not possible to determine the proportion of early *versus* delayed procedures in routine clinical practice, as the cohort exclusively included patients undergoing DC; however, in routine clinical practice, delayed procedures are expected to greatly outnumber early ones. Furthermore, as the study included only tertiary centres, the results may not be fully generalizable to secondary hospitals, although waiting list patterns are broadly similar across Spain^10^. Finally, beyond the hospitalized cohort analysed, a large proportion of patients with SGD were managed exclusively as outpatients and were therefore not captured in this study. Their trajectories through the waiting list and their risk of relapse remain poorly defined, despite being subject to the same structural limitations in surgical capacity. Future studies should specifically evaluate this outpatient population, as understanding its clinical course may further improve prioritization strategies and reduce overall delays.

Despite these limitations, the study offers notable strengths. With a large representative sample and a multicentre design, it has explored determinants of surgical waiting time within a national public health system in which delays are endemic.

Overall, the study has revealed excessively prolonged delays with clear clinical consequences. Importantly, the factors associated with shorter waiting times only partially aligned with established predictors of relapse, suggesting that current prioritization practices are not fully optimized from a clinical risk perspective. Failure to integrate such data-driven insights may perpetuate inefficiency and avoidable morbidity in already overstretched surgical systems. These findings should inform not only national prioritization frameworks but also broader efforts to improve elective surgical care across public health contexts.

## Supplementary Material

zrag021_Supplementary_Data

## Data Availability

The data that support the findings of this study are available from the corresponding author upon reasonable request.
