# Supplementary material for: Waiting for cholecystectomy: determinants of prioritization for delayed cholecystectomy in a universal public healthcare system—post hoc analysis of RELAPSTONE cohort (WAIT-CHOL study)
Source: BJS Open. 2026 Apr 15;10(2):zrag021. doi: 10.1093/bjsopen/zrag021 (PMC13080358; doi:10.1093/bjsopen/zrag021)
Supplement: zrag021_Supplementary_Data [file zrag021_supplementary_data.docx]

**Waiting for Cholecystectomy: Determinants of Prioritization for Delayed Cholecystectomy in a Universal Public Healthcare System – a Post-hoc Analysis of the RELAPSTONE Cohort (The WAIT-CHOL Study)**

*Raúl Velamazán^1,2,3^ - *Daniel Oyón^4^, Juan Lerma-Irureta^2^, Pablo López-Guillén^5^, Samuel J. Martínez-Domínguez^1,2,3^, Anna Arnau^,6,7,8^, Daniel Abad Baroja^2,9^, Lara M. Ruiz-Belmonte^1^, Javier Tejedor-Tejada^10^, Raul Zapater^11^, Noelia Martín-Vicente^12^, Pedro José Fernández-Esparcia^13^, Ana Belén Julián Gomara^9^, Violeta Sastre Lozano^14^, Juan José Manzanares García^14^, Irene Chivato Martín-Falquina^15^, Laura Andrés Pascual^15^, Nuria Torres Monclus^16^, Natividad Zaragoza Velasco^16^, Eukene Rojo^17,18^, Pablo Cañamares-Orbís^2,19^, Laura Pardo Grau^20^, María Vaamonde Lorenzo^21^, Arantzazu Izagirre Arostegi^21^, Virginia Flores^4^, Arantxa Diaz Gomez^4^, Ana Garcia de Paredes^3,11,22,23,^, Berta Lapeña‐Muñoz^24^, Guillermo García-Rayado^1,2^, Judith Millastre Bocos^1,2^, Vicente Borrego^25^, Carlos Sostres^1,2,3^, Angel Lanas^1,2,3^, Jose Manuel Ramia^26^, Enrique de-Madaria^27,28^

*Shared co-authorship.

^1^Department of Gastroenterology, Hospital Clínico Universitario Lozano Blesa, Zaragoza, Spain, ^2^IIS (Instituto de Investigacion Sanitaria) Aragón, Zaragoza,Spain, ^3^Centro de Investigacion Biomedica en Red de Enfermedades Hepaticas y Digestivas (CIBERehd) Instituto de Salud Carlos III, Madrid, Spain, ^4^Department of Gastroenterology. Hospital General Universitario Gregorio Marañón, Madrid, Spain. Instituto de Investigación Sanitaria Gregorio Marañón, Madrid, Spain ^5^Department of Gastroenterology, Hospital Universitario de Torrevieja, Alicante, Spain, ^6^Research and Innovation Unit, Althaia Xarxa Assistencial Universitària de Manresa, Manresa, Spain, ^7^Central Catalonia Chronicity Research Group (C3RG), Centre for Health and Social Care Research (CESS), University of Vic-Central University of Catalonia (UVIC-UCC), Vic, Spain, ^8^Faculty of Medicine, University of Vic-Central University of Catalonia (UVIC-UCC), Vic, Spain,^9^Department of Gastroenterology, Hospital Universitario Miguel Servet, Zaragoza, Spain ^10^Department of Gastroenterology, Hospital Universitario Río Hortega, Valladolid, Spain, ^11^Department of Gastroenterology and Hepatology, Hospital Universitario Ramón y Cajal, Madrid, Spain, ^12^ Department of Gastroenterology, Hospital de Galdakao, Bizkaia, Spain ^13^Miguel Hernandez University, Elche, Spain, ^14^Department of Gastroenterology, Hospital Universitario Santa Lucia, Cartagena, Spain, ^15^Department of Gastroenterology, Hospital Universitario de Burgos, Burgos, Spain, ^16^Department of Gastroenterology, Hospital Universitario Arnau de Vilanova, Lleida, Spain, ^17^Department of Gastroenterology, Hospital Universitario de La Princesa, Madrid, Spain, ^18^IIS (Instituto de Investigación Sanitaria)-Princesa, Madrid, Spain, ^19^Gastroenterology, Hepatology and Nutrition Unit, Hospital Universitario San Jorge, Huesca, Spain, Hospital Universitario San Jorge, Huesca, Spain, ^20^Department of Gastroenterology, Hospital Universitario Josep Trueta, Girona, Spain, ^21^Department of Gastroenterology, Hospital Universitario Donostia, Donostia, Spain, ^22^Universidad de Alcalá, Madrid, Spain, ^23^IRYCIS (Instituto Ramón y Cajal de Investigación Sanitaria), Madrid, Spain^,^, ^24^Department of Gastroenterology, Hospital Universitario San Pedro, Logroño, Spain. ^25^Department of surgery, Hospital Clínico Universitario Lozano Blesa, Zaragoza, Spain, ^26^Department of surgery, Hospital General Universitario Dr. Balmis, Alicante, Spain, ^27^Department of Gastroenterology, Hospital General Universitario Dr. Balmis-ISABIAL, Alicante, Spain ^28^ Department of Clinical Medicine, Miguel Hernandez University, Elche.

**Corresponding author**: Raúl Velamazán. Department of Gastroenterology, Hospital Clínico Universitario Lozano Blesa. San Juan Bosco, 15, 50009. Zaragoza, Spain.

**ORCID**: <https://orcid.org/0000-0002-2238-4418>. **Twitter**: @raulvelamazan

**Supplementary Material – Index**

[**Supplementary Methods** 3](#_Toc219202641)

[Participating centers 4](#_Toc219202642)

[Definitions 4](#_Toc219202643)

[Acute Pancreatitis (AP) 4](#_Toc219202644)

[Calculous acute cholecystitis (ACC) 4](#_Toc219202645)

[Acute calculous cholangitis (ACL) 5](#_Toc219202646)

[Symptomatic choledocholithiasis (SC) 6](#_Toc219202647)

[Biliary colic (BC) 6](#_Toc219202648)

[Any combination (Comb) 6](#_Toc219202649)

[Data collected 6](#_Toc219202650)

[Participants 10](#_Toc219202651)

[Supplementary Figure 1. 10](#_Toc219202652)

[**Supplementary Results**. 12](#_Toc219202653)

[Supplementary Table 1. 12](#_Toc219202654)

[Supplementary Table 2 17](#_Toc219202655)

[Supplementary Table 3. 22](#_Toc219202656)

[**References** 23](#_Toc219202657)

# Supplementary Methods

## Participating centers

Patients were recruited at 16 tertiary Spanish centers: University Hospital Lozano Blesa, Zaragoza; University Hospital Miguel Servet, Zaragoza; General University Hospital Dr. Balmis, Alicante; University Hospital Ramon y Cajal, Madrid. University Hospital Gregorio Marañon, Madrid; University Hospital La Princesa, Madrid; University Hospital Son Espases, Palma de Mallorca; University Hospital Santa Lucia, Cartagena; University Hospital Galdakao, Vizcaya; University Hospital San Pedro, Logroño; University Hospital of Donostia, Donostia; University Hospital of Burgos, Burgos; University Hospital Arnau de Vilanova, Lleida; University Hospital San Jorge, Huesca; University Hospital Josep Trueta, Girona; University Hospital Rio Hortega, Valladolid.

## Definitions

### Acute Pancreatitis (AP)

The definition of acute pancreatitis was based on: *Classification of acute pancreatitis-2012: revision of the Atlanta classification and definitions by international consensus*.^(1)^

The diagnosis of acute pancreatitis requires two of the following three features:

- Abdominal pain consistent with acute pancreatitis (acute onset of a persistent, severe, epigastric pain often radiating to the back).
- Serum lipase activity (or amylase activity) at least three times greater than the upper limit of normal.
- Characteristic findings of acute pancreatitis on contrast-enhanced computed tomography and less commonly magnetic resonance imaging or transabdominal ultrasonography.

To classify AP as biliary etiology, the presence of gallstones must be demonstrated by imaging test.

### Calculous acute cholecystitis (ACC)

The definition of acute cholecystitis was based on: the *Tokyo Guidelines 2018: diagnostic criteria and severity grading of acute cholecystitis*.^(2)^

The criteria to diagnose acute cholecystitis are divided into three categories: local signs of inflammation, systemic signs of inflammation, and imaging findings. A suspected diagnosis requires the presence of at least one item from category A and one item from category B. A definite diagnosis requires the presence of at least one item from category A, one item from category B, and imaging findings (category C).

A. Local signs of inflammation:

- Murphy's sign.

- Presence of a mass, pain, or tenderness in the right upper quadrant of the abdomen.

B. Systemic signs of inflammation:

- Fever.

- Elevated C-reactive protein.

- Elevated white blood cells in the blood.

C. Imaging findings characteristic of acute cholecystitis and presence of gallstones

### Acute calculous cholangitis (ACL)

The definition of acute calculous cholangitis was based on: the *Tokyo Guidelines 2018: diagnostic criteria and severity grading of acute cholangitis*.^(3)^

The criteria are divided into three categories: systemic inflammation, cholestasis, and imaging. A suspected diagnosis requires the presence of at least one item from category A and one item from either category B or category C. A definite diagnosis requires the presence of at least one item from category A, one item from category B, and one item from category C.

A. Systemic inflammation:

- Fever and/or shaking chills.

- Laboratory data: evidence of inflammatory response such as elevated levels of inflammatory markers like C-reactive protein or an increased white blood cell count.

B. Cholestasis:

- Jaundice: Total bilirubin ≥2 (mg/dl).

- Laboratory data: abnormal liver function tests such as elevated levels of bilirubin, liver enzymes or alkaline phosphatase.

C. Imaging:

- Biliary dilatation.

- Presence of gallstones

###

### Symptomatic choledocholithiasis (SC)

We define symptomatic choledocholithiasis as the symptomatology secondary to the presence of a common duct stone confirmed by imaging tests, and in the absence of criteria for acute cholangitis or acute pancreatitis.^(4)^

### Biliary colic (BC)

Defined as the typical biliary pain: epigastric with radiation to the right upper quadrant, intense, of short duration (<6 hours), occasionally accompanied by nausea or vomiting, in a patient with visualized cholelithiasis on imaging tests and in the absence of criteria for choledocholithiasis or other SGD.^(4)^

### Any combination (Comb)

If a patient presented criteria of several diseases at the same time, the case was labeled under the any combination group, thus forming a miscellaneous group with patients who could have a combination of acute pancreatitis, acute cholecystitis, acute cholangitis and/or choledocholithiasis.

## Data collected

The following data were collected:

- Demographics:

- Age, gender.

- Habits:

- Tobacco, and alcohol use.

- Comorbidity:

- Charlson comorbidity index (takes into account the presence of myocardial infarction, congestive heart failure, cerebrovascular disease, dementia, chronic pulmonary disease, rheumatologic disease, peptic ulcer disease, mild liver disease, moderate or severe liver disease, diabetes with and without chronic complications, hemiplegia or paraplegia, renal disease, any non metastatic solid tumor, metastatic solid tumor, leukemia, lymphoma, AIDS).^(5)^

- Type and characteristics of gallstone symptomatic disease that cause index admission: AP, ACC, ACL, SC, BC and comb.

- Severity index was calculated according to the Atlanta classification for AP.^(1)^
  - Mild acute pancreatitis: characterized by the absence of organ failure and the absence of local or systemic complications.
  - Moderately severe acute pancreatitis: characterized by the presence of transient (<48h) organ failure or local or systemic complications in the absence of persistent organ failure.
  - Severe acute pancreatitis: characterized by persistent (>48h) organ failure.
- Severity index was calculated according to the Tokyo classification for ACC.^(2)^
  - Grade III (severe) acute cholecystitis: ACC associated with dysfunction of any one of the following organs/systems:
    - Cardiovascular dysfunction: hypotension requiring treatment with dopamine ≥5 µg/kg per min, or any dose of norepinephrine.
    - Neurological dysfunction: decreased level of consciousness.
    - Respiratory dysfunction: PaO2/FiO2 ratio <300.
    - Renal dysfunction: oliguria, creatinine >2.0 mg/dl.
    - Hepatic dysfunction: PT-INR >1.5.
    - Hematological dysfunction: platelet count <100,000/mm3.
  - Grade II (moderate) acute cholecystitis: ACC associated with any one of the following conditions:
    - Elevated WBC count (>18,000/mm3).
    - Palpable tender mass in the right upper abdominal quadrant.
    - Duration of complaints >72 h.
    - Marked local inflammation (gangrenous cholecystitis, pericholecystic abscess, hepatic abscess, biliary peritonitis, emphysematous cholecystitis).
  - Grade I (mild) acute cholecystitis “ACC that does not meet the criteria of “Grade III” or “Grade II” acute cholecystitis.
- Severity index was calculated according to the Tokyo classification for ACL.^(3)^
  - Grade III (severe) acute cholangitis “ACL that is associated with the onset of dysfunction at least in any one of the following organs/systems:
    - Cardiovascular dysfunction: hypotension requiring dopamine ≥5 µg/kg per min, or any dose of norepinephrine.
    - Neurological dysfunction: disturbance of consciousness.
    - Respiratory dysfunction: PaO2/FiO2 ratio <300.
    - Renal dysfunction: oliguria, serum creatinine >2.0 mg/dl.
    - Hepatic dysfunction: PT-INR >1.5.
    - Hematological dysfunction: platelet count <100,000/mm3.
  - Grade II (moderate) acute cholangitis: ACL associated with any two of the following conditions:
    - Abnormal WBC count (>12,000/mm3, <4,000/mm3).
    - High fever (≥39°C).
    - Age (≥75 years old).
    - Hyperbilirubinemia (total bilirubin ≥5 mg/dl).
    - Hypoalbuminemia (<STD x 0.7).
  - Grade I (mild) acute cholangitis: ACL that does not meet the criteria of “Grade III” or “Grade II” ACL at initial diagnosis.
- Presence of: acute renal dysfunction (Creatinine ≥ 1,9mg/dL), acute respiratory dysfunction (PaO2/FiO2 < 300 or tracheal intubation), or acute cardiovascular dysfunction ([systolic pressure](https://www.google.com/search?sxsrf=ALiCzsZTWyuls_rAG_bxCPiDAYgp1pjang:1671788637068&q=systolic+pressure&spell=1&sa=X&ved=2ahUKEwjVkej-uY_8AhUtu6QKHS6pDE8QkeECKAB6BAgIEAE) < 90mmHg despite the use of fluidotherapy).^(1)^
- Blood and bile cultures.
- ICU admission and length of stay.
- Presence of fluid or necrotic pancreatic, peripancreatic or pericholecystic collection and liver abscess.^(1)^
- Use of complementary exams (transabdominal ultrasonography, computed axial tomography (CT), magnetic resonance imaging (RMI), endoscopic ultrasound (EUS)).
- Characteristics of cholelithiasis: absence, microlithiasis/biliary sludge, unique cholelithiasis, multiple cholelithiases, or indeterminate.
- Characteristics of the biliary tract in the most reliable complementary test (being RMI or EUS the most reliable followed by CT and transabdominal ultrasound): non dilated, isolated extra-hepatic dilatation, isolated intra-hepatic dilatation, both intra and extra-hepatic dilatation or dilated but not determined which tract.
- Diameter of the dilated biliary tract.
- Presence of duodenal diverticula or pancreas divisum (considered only when CT, MRI, EUS, or ERCP were performed, otherwise patient count as invalid.)
- ERCP: performance of sphincterotomy, Wirsung cannulation, placing Wirsung stent, successful ERCP with regards to eliminating all choledocholithiasis in the same ERCP, placing biliary sent, and presence of complications.
- PTHC: placing biliary stent, successful PTHC with regards to eliminating all choledocholithiasis in the same PTHC, and presence of complications.
- Cholecystostomy: the way to perform it (percutaneous, EUS guided), and presence of complications.
- Commencement of treatment with Ursodeoxycholic acid at discharge.
- Length of hospital stay (days).
- Measurement of analytic parameters at three different moments during hospital admission: at admission (first 24 hours), at discharge (in the last 48 hours of the admission), and the highest value of the parameter during admission. The following parameters were collected: [aspartate transaminase](https://en.wikipedia.org/wiki/Aspartate_transaminase) (U/L), [alanine transaminase](https://en.wikipedia.org/wiki/Alanine_transaminase) (U/L), gamma-glutamyl transferase (U/L), alkaline phosphatase (U/L), bilirubin (mg/dl), leukocytes (/mm^3^), neutrophils (/mm^3^), lymphocytes (/mm^3^), hematocrit (%), urea (mg/dl), and C-reactive protein (mg/L). The pathological cut-off value for each parameter was: [aspartate transaminase](https://en.wikipedia.org/wiki/Aspartate_transaminase): 35U/L, [alanine transaminase](https://en.wikipedia.org/wiki/Alanine_transaminase): 35U/L, gamma-glutamyl transferase: 78 U/L, alkaline phosphatase: 150U/L, bilirubin: 1,2 mg/dl, leucocytes: 11000/mm^3^, neutrophils: 8500/mm^3^, lymphocytes: 4500/mm^3^, hematocrit: 47% for females and 51% for males, urea: 54mg/dl, and C-reactive protein: 5mg/L.

- Presence and recount of relapses and their characteristics: date, type of SGD, severity, and need for hospital admission.

- Mortality and date during follow-up.

- Characteristics of cholecystectomy and date during follow-up.

## Participants

Of the 3,016 patients included in the RELAPSTONE study, 1,572 underwent delayed cholecystectomy. From this group, we excluded 52 patients from Mexican centers to focus exclusively on the Spanish population, as well as 12 patients with missing surgery dates. The final study population comprised 1,508 patients. (Supplementary Figure 1)

## Supplementary Figure 1.

Assessed for eligibility: n=3016

Met one or more exclusion criteria: n=1496 (49.6%)

- No cholecystectomy performed:1444
- Non-Spanish hospitals: 52

n=1520

Lack of information about date of cholecystectomy: n=12 (0.4%)

n=1508

**Supplementary Figure 1**. Flowchart showing patient screening for eligibility and subsequent exclusions leading to the final study population.

# Supplementary Results.

Supplementary Table 1. Baseline sample characteristics, median waiting time to cholecystectomy, distribution of cholecystectomies by waiting time intervals (<3, 3–6, and >6 months) and bivariate analysis. N=1508.

|  | N Valid | N (%) | Median waiting time to cholecystectomy (months) | <3 months  N (%) | 3-6 months  N (%) | >6 months  N (%) | β | 95% CI β | Unadjusted p-value | Unadjusted Model  p-value |
| --- | --- | --- | --- | --- | --- | --- | --- | --- | --- | --- |
| Tobacco | 1158 |  |  |  |  |  |  |  |  | 0.212 |
| Never |  | 722 (62.3%) | 4.2 (2.3 - 6.6) | 247 (34.2%) | 255 (35.3%) | 220 (30.5%) | 0.0 |  |  |  |
| Ex-smoking |  | 248 (21.4%) | 4.9 (2.5 - 7.4) | 71 (28.6%) | 83 (33.5%) | 94 (37.9%) | 0.5 | -0.1 - 1.2 | 0.103 |  |
| Active-smoking |  | 188 (16.3%) | 4.5 (2.2 - 7.1) | 63 (33.5%) | 53 (28.2%) | 72 (38.3%) | 0.4 | -0.3 - 1.1 | 0.305 |  |
| Alcohol | 1094 |  |  |  |  |  |  |  |  | < 0.001 |
| Never |  | 836 (76.4%) | 4.2 (2.3 - 6.7) | 291 (34.8%) | 282 (33.7%) | 263 (31.5%) | 0.0 |  |  |  |
| Ex-use |  | 37 (3.4%) | 5.3 (2.4 - 8.0) | 10 (27%) | 10 (27%) | 17 (45.9%) | 3.0 | 1.5 - 4.4 | < 0.001 |  |
| Active use |  | 221 (20.2%) | 4.8 (2.8 - 7.1) | 61 (27.6%) | 78 (35.3%) | 82 (37.1%) | 0.6 | -0.1 - 1.3 | 0.060 |  |
| Charlson comorbidity index diseases | | |  |  |  |  |  |  |  |  |
| Myocardial infarction | 1508 |  |  |  |  |  |  |  |  | 0.251 |
| No |  | 1402 (92.8%) | 4.4 (2.3 - 6.9) | 477 (34%) | 443 (31.6%) | 482 (34.4%) | 0.0 |  |  |  |
| Yes |  | 106 (7.0%) | 4.7 (2.5 - 7.1) | 32 (30.2%) | 34 (32.1%) | 40 (37.7%) | 0.5 | -0.4 - 1.4 |  |  |
| Congestive heart failure | 1508 |  |  |  |  |  |  |  |  | 0.299 |
| No |  | 1445 (95.6%) | 4.4 (2.3 - 6.9) | 491 (34%) | 459 (31.8%) | 495 (34.3%) | 0.0 |  |  |  |
| Yes |  | 63 (4.2%) | 4.9 (2.5 - 8.4) | 18 (28.6%) | 18 (28.6%) | 27 (42.9%) | 0.6 | -0.5 - 1.7 |  |  |
| Peripheral vascular disease | 1508 |  |  |  |  |  |  |  |  | 0.228 |
| No |  | 1458 (96.5%) | 4.4 (2.3 - 6.9) | 495 (34%) | 462 (31.7%) | 501 (34.4%) | 0.0 |  |  |  |
| Yes |  | 50 (3.3%) | 5.1 (2.8 - 7.3) | 14 (28%) | 15 (30%) | 21 (42%) | 0.8 | -0.5 - 2.1 |  |  |
| Cerebrovascular disease | 1508 |  |  |  |  |  |  |  |  | 0.722 |
| No |  | 1434 (94.9%) | 4.5 (2.3 – 7.0) | 485 (33.8%) | 453 (31.6%) | 496 (34.6%) | 0.0 |  |  |  |
| Yes |  | 74 (5.1%) | 4.3 (2.4 - 7.1) | 24 (32.4%) | 24 (32.4%) | 26 (35.1%) | -0.2 | -1.2 - 0.9 |  |  |
| Dementia | 1508 |  |  |  |  |  |  |  |  | 0.461 |
| No |  | 1484 (98.2%) | 4.4 (2.3 - 6.9) | 504 (34%) | 473 (31.9%) | 507 (34.2%) | 0.0 |  |  |  |
| Yes |  | 24 (1.6%) | 6.4 (3.2 - 8.6) | 5 (20.8%) | 4 (16.7%) | 15 (62.5%) | 0.7 | -1.1 - 2.5 |  |  |
| Chronic pulmonary disease | 1508 |  |  |  |  |  |  |  |  | 0.394 |
| No |  | 1353 (89.5%) | 4.4 (2.3 - 6.9) | 458 (33.9%) | 429 (31.7%) | 466 (34.4%) | 0.0 |  |  |  |
| Yes |  | 155 (10.3%) | 4.6 (2.3 - 7.2) | 51 (32.9%) | 48 (31%) | 56 (36.1%) | 0.3 | -0.4 - 1.1 |  |  |
| Rheumatologic disease | 1508 |  |  |  |  |  |  |  |  | 0.362 |
| No |  | 1468 (97.2%) | 4.4 (2.3 - 6.9) | 499 (34%) | 462 (31.5%) | 507 (34.5%) | 0.0 |  |  |  |
| Yes |  | 40 (2.7%) | 5.3 (3.1 - 7.1) | 10 (25%) | 15 (37.5%) | 15 (37.5%) | 0.7 | -0.8 - 2.1 |  |  |
| Peptic ulcer disease | 1508 |  |  |  |  |  |  |  |  | 0.509 |
| No |  | 1446 (95.7%) | 4.5 (2.3 – 7.0) | 486 (33.6%) | 461 (31.9%) | 499 (34.5%) | 0.0 |  |  |  |
| Yes |  | 62 (4.1%) | 4.5 (2.1 - 6.7) | 23 (37.1%) | 16 (25.8%) | 23 (37.1%) | -0.4 | -1.5 - 0.8 |  |  |
| Diabetes | 1508 |  |  |  |  |  |  |  |  | 0.613 |
| No |  | 1197 (79.2%) | 4.5 (2.3 - 6.9) | 406 (33.9%) | 378 (31.6%) | 413 (34.5%) | 0.0 |  |  |  |
| Without chronic complications |  | 280 (18.5%) | 4.5 (2.4 - 7.5) | 91 (32.5%) | 88 (31.4%) | 101 (36.1%) | 0.3 | -0.3 - 0.9 | 0.387 |  |
| With chronic complications |  | 31 (2.1%) | 3.4 (2.0- 6.0) | 12 (38.7%) | 11 (35.5%) | 8 (25.8%) | -0.3 | -2.0 - 1.3 | 0.677 |  |
| Hemiplegia or paraplegia | 1507 |  |  |  |  |  |  |  |  | 0.198 |
| No |  | 1499 (99.2%) | 4.4 (2.3 – 7.0) | 507 (33.8%) | 474 (31.6%) | 518 (34.6%) | 0.0 |  |  |  |
| Yes |  | 8 (0.5%) | 5.1 (3.4 - 6.2) | 2 (25%) | 3 (37.5%) | 3 (37.5%) | 2.1 | -1.1 - 5.2 |  |  |
| Chronic renal disease | 1508 |  |  |  |  |  |  |  |  | 0.834 |
| No |  | 1425 (94.3%) | 4.4 (2.3 - 6.9) | 484 (34%) | 454 (31.9%) | 487 (34.1%) | 0.0 |  |  |  |
| Yes |  | 83 (5.5%) | 4.9 (2.5 - 7.2) | 25 (30.1%) | 23 (27.7%) | 35 (42.2%) | 0.1 | -0.9 - 1.1 |  |  |
| Solid tumor | 1508 |  |  |  |  |  |  |  |  | 0.283 |
| No |  | 1365 (90.3%) | 4.4 (2.3 – 7.0) | 463 (33.9%) | 427 (31.3%) | 475 (34.8%) | 0.0 |  |  |  |
| Nor metastasic |  | 135 (8.9%) | 4.6 (2.4 - 6.9) | 42 (31.1%) | 48 (35.6%) | 45 (33.3%) | 0.2 | -0.6 – 1.0 | 0.672 |  |
| Metastatic |  | 8 (0.5%) | 2.3 (1.3 - 4.3) | 4 (50%) | 2 (25%) | 2 (25%) | -2.4 | -5.6 - 0.7 | 0.128 |  |
| Leukemia | 1508 |  |  |  |  |  |  |  |  | 0.491 |
| No |  | 1502 (99.4%) | 4.5 (2.3 – 7.0) | 506 (33.7%) | 475 (31.6%) | 521 (34.7%) | 0.0 |  |  |  |
| Yes |  | 6 (0.4%) | 2.7 (1.8 - 3.9) | 3 (50%) | 2 (33.3%) | 1 (16.7%) | -1.3 | -4.9 - 2.3 | 0.491 |  |
| Lymphoma | 1508 |  |  |  |  |  |  |  |  | 0.499 |
| No |  | 1499 (99.2%) | 4.4 (2.3 – 7.0) | 508 (33.9%) | 474 (31.6%) | 517 (34.5%) | 0.0 |  |  |  |
| Yes |  | 9 (0.6%) | 6.1 (4.2 - 7.1) | 1 (11.1%) | 3 (33.3%) | 5 (55.6%) | 1.0 | -1.9 – 4.0 | 0.499 |  |
| AIDS | 1508 |  |  |  |  |  |  |  |  | 0.998 |
| No |  | 1507 (99.7%) | 4.4 (2.3 – 7.0) | 509 (33.8%) | 476 (31.6%) | 522 (34.6%) | 0.0 |  |  |  |
| Yes |  | 1 (0.07%) | 5.4 | 0 (0%) | 1 (100%) | 0 (0%) | 0.0 | -8.9 - 8.8 | 0.998 |  |
| Acute renal dysfunction | 1506 |  |  |  |  |  |  |  |  | 0.183 |
| No |  | 1447 (96.1%) | 4.4 (2.3 - 6.9) | 490 (33.9%) | 462 (31.9%) | 495 (34.2%) | 0.0 |  |  |  |
| Yes |  | 59 (3.9%) | 5.3 (2.2 - 7.4) | 18 (30.5%) | 15 (25.4%) | 26 (44.1%) | 0.8 | -0.4 – 2.0 |  |  |
| Acute respiratory dysfunction | 1507 |  |  |  |  |  |  |  |  | 0.012 |
| No |  | 1489 (98.8%) | 4.4 (2.3 - 6.9) | 504 (33.8%) | 474 (31.8%) | 511 (34.3%) | 0.0 |  |  |  |
| Yes |  | 18 (1.2%) | 6.5 (3.1 - 13.4) | 5 (27.8%) | 3 (16.7%) | 10 (55.6%) | 2.7 | 0.6 - 4.8 |  |  |
| Acute cardiovascular dysfunction | 1508 |  |  |  |  |  |  |  |  | 0.258 |
| No |  | 1480 (98.1%) | 4.5 (2.3 – 7.0) | 495 (33.4%) | 473 (32%) | 512 (34.6%) | 0.0 |  |  |  |
| Yes |  | 28 (1.9%) | 3.3 (1.3 - 6.3) | 14 (50%) | 4 (14.3%) | 10 (35.7%) | -1.0 | -2.7 - 0.7 |  |  |
| Positive blood cultures | 1508 |  |  |  |  |  |  |  |  | 0.306 |
| No |  | 1411 (93.6%) | 4.4 (2.3 - 6.9) | 477 (33.8%) | 450 (31.9%) | 484 (34.3%) | 0.0 |  |  |  |
| Yes |  | 97 (6.4%) | 4.6 (2.2 - 8.2) | 32 (33%) | 27 (27.8%) | 38 (39.2%) | 0.5 | -0.4 - 1.4 |  |  |
| Positive bile cultures | 1508 |  |  |  |  |  |  |  |  | 0.026 |
| No |  | 1450 (96.2%) | 4.5 (2.3 – 7.0) | 479 (33%) | 465 (32.1%) | 506 (34.9%) | 0.0 |  |  |  |
| Yes |  | 58 (3.9%) | 2.9 (1.7 - 6.2) | 30 (51.7%) | 12 (20.7%) | 16 (27.6%) | -1.3 | -2.5 - -0.2 |  |  |
| ICU admission | 1508 |  |  |  |  |  |  |  |  | 0.516 |
| No |  | 1484 (98.4%) | 4.4 (2.3 - 6.9) | 501 (33.8%) | 473 (31.9%) | 510 (34.4%) | 0.0 |  |  |  |
| Yes |  | 24 (1.6%) | 5.7 (2.0 - 8.7) | 8 (33.3%) | 4 (16.7%) | 12 (50%) | 0.6 | -1.2 - 2.4 |  |  |
| Any pancreatic collection | 1508 |  |  |  |  |  |  |  |  | 0.067 |
| No |  | 1379 (91.5%) | 4.4 (2.3 - 6.9) | 473 (34.3%) | 440 (31.9%) | 466 (33.8%) | 0.0 |  |  |  |
| Yes |  | 129 (8.5%) | 5.2 (2.9 - 7.6) | 36 (27.9%) | 37 (28.7%) | 56 (43.4%) | 0.8 | -0.1 - 1.6 |  |  |
| Acute peripancreatic fluid collection or pancreatic pseudocyst | 1508 |  |  |  |  |  |  |  |  | 0.926 |
| No |  | 1440 (95.5%) | 4.5 (2.3 - 6.9) | 483 (33.5%) | 462 (32.1%) | 495 (34.4%) | 0.0 |  |  |  |
| Yes |  | 68 (4.5%) | 4.6 (2.3 - 7.2) | 26 (38.2%) | 15 (22.1%) | 27 (39.7%) | -0.1 | -1.2 - 1.0 |  |  |
| Pericholecystic fluid collections | 1508 |  |  |  |  |  |  |  |  | 0.367 |
| No |  | 1440 (95.5%) | 4.5 (2.3 – 7.0) | 481 (33.4%) | 462 (32.1%) | 497 (34.5%) | 0.0 |  |  |  |
| Yes |  | 68 (4.5%) | 3.8 (2.0 – 7.0) | 28 (41.2%) | 15 (22.1%) | 25 (36.8%) | -0.5 | -1.6 - 0.6 |  |  |
| Hepatic abscess | 1507 |  |  |  |  |  |  |  |  | 0.380 |
| No |  | 1489 (98.8%) | 4.5 (2.3 – 7.0) | 502 (33.7%) | 470 (31.6%) | 517 (34.7%) | 0.0 |  |  |  |
| Yes |  | 18 (1.2%) | 3.7 (2.3 - 6.4) | 7 (38.9%) | 6 (33.3%) | 5 (27.8%) | -0.9 | -3.0 - 1.2 |  |  |
| Biliary tract | 1508 |  |  |  |  |  |  |  |  | 0.124 |
| Not dilatated |  | 1043 (69.2%) | 4.4 (2.4 - 6.9) | 350 (33.6%) | 340 (32.6%) | 353 (33.8%) | 0.0 |  |  |  |
| Dilated |  | 465 (30.8%) | 4.6 (2.3 - 7.1) | 159 (34.2%) | 137 (29.5%) | 169 (36.3%) | 0.4 | -0.1 - 0.9 |  |  |
| ERCP | 1508 |  |  |  |  |  |  |  |  | 0.922 |
| No |  | 1139 (75.5%) | 4.5 (2.4 – 7.1) | 372 (32.7%) | 369 (32.4%) | 398 (34.9%) | 0.0 |  |  |  |
| Yes |  | 369 (24.5%) | 4.2 (2.2 - 6.9) | 137 (37.1%) | 108 (29.3%) | 124 (33.6%) | 0.0 | -0.6 - 0.5 |  |  |
| PTHC | 1508 |  |  |  |  |  |  |  |  | 0.656 |
| No |  | 1506 (99.9%) | 4.5 (2.3 – 7.0) | 508 (33.7%) | 477 (31.7%) | 521 (34.6%) | 0.0 |  |  |  |
| Yes |  | 2 (0.1%) | 6.9 (3.8 - 9.9) | 1 (50%) | 0 (0%) | 1 (50%) | 1.4 | -4.8 - 7.7 | 0.656 |  |
| Cholecystostomy | 1506 |  |  |  |  |  |  |  |  | 0.036 |
| No |  | 1425 (94.6%) | 4.5 (2.4 – 7.0) | 466 (32.7%) | 462 (32.4%) | 497 (34.9%) | 0.0 |  |  |  |
| Percutaneous |  | 78 (5.2%) | 2.8 (1.7 - 6.6) | 41 (52.6%) | 14 (17.9%) | 23 (29.5%) | -1.4 | -2.4- -0.3 | 0.010 |  |
| EUS-guided |  | 3 (0.2%) | 1.4 (1.4 - 7.1) | 2 (66.7%) | 0 (0%) | 1 (33.3%) | -0.3 | -5.4 - 4.8 | 0.910 |  |
| Commencement of UDCA at discharge | 1508 |  |  |  |  |  |  |  |  | 0.526 |
| No |  | 1490 (98.8%) | 4.5 (2.3 – 7.0) | 503 (33.8%) | 470 (31.5%) | 517 (34.7%) | 0.0 |  |  |  |
| Yes |  | 18 (1.2%) | 4.6 (2.6 - 6.1) | 6 (33.3%) | 7 (38.9%) | 5 (27.8%) | -0.7 | -2.3 - 1.4 |  |  |

Data presented as: n (%); median (25th percentile–75th percentile).

β: β coefficient; CI: Confidence Interval; AIDS: acquired immunodeficiency syndrome; ICU: Intensive care unit; ERCP: Endoscopic Retrograde Cholangiopancreatography; PTHC: Percutaneous Transhepatic Cholangiogram; EUS: Endoscopic ultrasound; UDCA: Ursodeoxycholic acid.

Supplementary Table 2**.** Analytical parameters, median waiting time to cholecystectomy, distribution of cholecystectomies by waiting time intervals (<3, 3–6, and >6 months) and bivariate analysis. N= 1508.

|  | N Valid | N (%) | Median waiting time to cholecystectomy (months) | <3 months  N (%) | 3-6 months  N (%) | >6 months  N (%) | β | 95% CI β | Unadjusted Model  p-value |
| --- | --- | --- | --- | --- | --- | --- | --- | --- | --- |
| AST at admission (U/L) | 1508 |  |  |  |  |  |  |  |  |
| ≤35 |  | 346 (23.1%) | 4.6 (2.3 - 6.9) | 111 (32.1%) | 122 (35.3%) | 113 (32.7%) | 0 |  |  |
| >35 |  | 1162 (76.9%) | 4.4 (2.3 – 7.0) | 398 (34.3%) | 355 (30.6%) | 409 (35.2%) | 0.1 | -0.4 - 0.7 | 0.697 |
| AST highest value (U/L) | 1508 |  |  |  |  |  |  |  |  |
| ≤35 |  | 262 (17.5%) | 4.6 (2.5 - 6.9) | 85 (32.4%) | 92 (35.1%) | 85 (32.4%) | 0 |  |  |
| >35 |  | 1246 (82.5%) | 4.4 (2.3 – 7.0) | 424 (34%) | 385 (30.9%) | 437 (35.1%) | 0.2 | -0.4 - 0.8 | 0.482 |
| AST at discharge (U/L) | 1508 |  |  |  |  |  |  |  |  |
| ≤35 |  | 807 (53.5%) | 4.5 (2.3 - 7.3) | 273 (33.8%) | 242 (30%) | 292 (36.2%) | 0 |  |  |
| >35 |  | 701 (46.5%) | 4.3 (2.3 - 6.7) | 236 (33.7%) | 235 (33.5%) | 230 (32.8%) | -0.4 | -0.8 - 0.1 | 0.096 |
| ALT at admission (U/L) | 1508 |  |  |  |  |  |  |  |  |
| ≤35 |  | 312 (20.7%) | 4.6 (2.4 - 6.9) | 105 (33.7%) | 101 (32.4%) | 106 (34%) | 0 |  |  |
| >35 |  | 1196 (79.3%) | 4.4 (2.3 – 7,0) | 404 (33.8%) | 376 (31.4%) | 416 (34.8%) | 0.1 | -0.4 - 0.7 | 0.684 |
| ALT at discharge (U/L) | 1508 |  |  |  |  |  |  |  |  |
| ≤35 |  | 440 (29.3%) | 4.6 (2.3- 7.5) | 143 (32.5%) | 131 (29.8%) | 166 (37.7%) | 0 |  |  |
| >35 |  | 1068 (70.7%) | 4.4 (2.3 - 6.8) | 366 (34.3%) | 346 (32.4%) | 356 (33.3%) | -0.4 | -0.9 - 0.1 | 0.095 |
| GGT at admission (U/L) | 1508 |  |  |  |  |  |  |  |  |
| ≤78 |  | 280 (18.6%) | 4.9 (2.5 - 7.0) | 91 (32.5%) | 87 (31.1%) | 102 (36.4%) | 0 |  |  |
| >78 |  | 1228 (81.4%) | 4.3 (2.3 - 6.9) | 418 (34%) | 390 (31.8%) | 420 (34.2%) | -0.1 | -0.6 - 0.6 | 0.909 |
| GGT highest value (U/L) | 1508 |  |  |  |  |  |  |  |  |
| ≤78 |  | 190 (12.6%) | 5.0 (2.4 - 7.1) | 59 (31.1%) | 58 (30.5%) | 73 (38.4%) | 0 |  |  |
| >78 |  | 1318 (87.4%) | 4.3 (2.3 - 6.9) | 450 (34.1%) | 419 (31.8%) | 449 (34.1%) | -0.2 | -0.9 - 0.5 | 0.515 |
| GGT at discharge (U/L) | 1508 |  |  |  |  |  |  |  |  |
| ≤78 |  | 260 (17.2%) | 5.2 (2.5 - 7.4) | 77 (29.6%) | 79 (30.4%) | 104 (40%) | 0 |  |  |
| >78 |  | 1248 (82.8%) | 4.3 (2.2 - 6.9) | 432 (34.6%) | 398 (31.9%) | 418 (33.5%) | -0.4 | -1.0 - 0.2 | 0.156 |
| ALP at admission (U/L) | 1508 |  |  |  |  |  |  |  |  |
| ≤150 |  | 898 (59.4%) | 4.6 (2.4 – 7.0) | 291 (32.4%) | 285 (31.7%) | 322 (35.9%) | 0 |  |  |
| >150 |  | 610 (40.6%) | 4.2 (2.2 – 7.0) | 218 (35.7%) | 192 (31.5%) | 200 (32.8%) | -0.2 | -0.6 - 0.3 | 0.474 |
| ALP highest value (U/L) | 1508 |  |  |  |  |  |  |  |  |
| ≤150 |  | 645 (42.7%) | 4.6 (2.4 - 6.9) | 212 (32.9%) | 206 (31.9%) | 227 (35.2%) | 0 |  |  |
| >150 |  | 863 (57.3%) | 4.3 (2.3 - 7.1) | 297 (34.4%) | 271 (31.4%) | 295 (34.2%) | 0.1 | -0.4 - 0.6 | 0.685 |
| ALP at discharge (U/L) | 1508 |  |  |  |  |  |  |  |  |
| ≤150 |  | 932 (61.7%) | 4.3 (2.4 - 6.9) | 315 (33.8%) | 304 (32.6%) | 313 (33.6%) | 0 |  |  |
| >150 |  | 576 (38.3%) | 4.7 (2.3 - 7.2) | 194 (33.7%) | 173 (30%) | 209 (36.3%) | 0.3 | -0.2 - 0.7 | 0.275 |
| Bilirrubin at admission (mg/dl) | 1508 |  |  |  |  |  |  |  |  |
| ≤1.2 |  | 560 (37.1%) | 4.5 (2.3 - 6.7) | 186 (33.2%) | 191 (34.1%) | 183 (32.7%) | 0 |  |  |
| >1.2 |  | 948 (62.9%) | 4.4 (2.3 - 7.1) | 323 (34.1%) | 286 (30.2%) | 339 (35.8%) | 0.1 | -0.4 - 0.6 | 0.704 |
| Bilirrubin highest value mg/dl) | 1508 |  |  |  |  |  |  |  |  |
| ≤1.2 |  | 487 (32.3%) | 4.6 (2.2 - 6.8) | 166 (34.1%) | 156 (32%) | 165 (33.9%) | 0 |  |  |
| >1.2 |  | 1021 (67.7%) | 4.4 (2.4 - 7.1) | 343 (33.6%) | 321 (31.4%) | 357 (35%) | 0.1 | -0.5 - 0.5 | 0.871 |
| Bilirrubin at discharge (mg/dl) | 1508 |  |  |  |  |  |  |  |  |
| ≤1.2 |  | 1131 (74.9%) | 4.4 (2.3 - 7.0) | 380 (33.6%) | 360 (31.8%) | 391 (34.6%) | 0 |  |  |
| >1.2 |  | 377 (25.1%) | 4.5 (2.3 - 6.9) | 129 (34.2%) | 117 (31%) | 131 (34.7%) | -0.1 | -0.6 - 0.4 | 0.726 |
| Leucocytes at admission count/mm^3^) | 1508 |  |  |  |  |  |  |  |  |
| ≤11000 |  | 810 (53.6%) | 4.0 (2.2 - 6.5) | 297 (36.7%) | 269 (33.2%) | 244 (30.1%) | 0 |  |  |
| >11000 |  | 698 (46.4%) | 5.0 (2.5 - 7.4) | 212 (30.4%) | 208 (29.8%) | 278 (39.8%) | 0.6 | 0.2 - 1.0 | 0.01 |
| Leucocytes at discharge (count/mm^3^) | 1508 |  |  |  |  |  |  |  |  |
| ≤11000 |  | 1356 (89.7%) | 4.4 (2.3 - 6.9) | 460 (33.9%) | 436 (32.2%) | 460 (33.9%) | 0 |  |  |
| >11000 |  | 152 (10.3%) | 5.1 (2.5 – 8.0) | 49 (32.2%) | 41 (27%) | 62 (40.8%) | 0.4 | -0.4 - 1.1 | 0.334 |
| Neutrophils at admission (count/mm^3^) | 1508 |  |  |  |  |  |  |  |  |
| ≤8500 |  | 764 (50.6%) | 4.2 (2.2 - 6.6) | 277 (36.3%) | 247 (32.3%) | 240 (31.4%) | 0 |  |  |
| >8500 |  | 744 (49.4%) | 4.8 (2.5 - 7.3) | 232 (31.2%) | 230 (30.9%) | 282 (37.9%) | 0.4 | -0.1 - 0.9 | 0.083 |
| Neutrophils highest value (count/mm^3^) | 1508 |  |  |  |  |  |  |  |  |
| ≤8500 |  | 663 (43.9%) | 4.3 (2.2 - 6.5) | 240 (36.2%) | 216 (32.6%) | 207 (31.2%) | 0 |  |  |
| >8500 |  | 845 (56.1%) | 4.7 (2.4 - 7.3) | 269 (31.8%) | 261 (30.9%) | 315 (37.3%) | 0.4 | -0.1 - 0.8 | 0.119 |
| Neutrophils at discharge (count/mm^3^) | 1508 |  |  |  |  |  |  |  |  |
| ≤8500 |  | 1400 (92.7%) | 4.4 (2.3 - 6.9) | 475 (33.9%) | 447 (31.9%) | 478 (34.1%) | 0 |  |  |
| >8500 |  | 108 (7.3%) | 5.0 (2.5 - 7.8) | 34 (31.5%) | 30 (27.8%) | 44 (40.7%) | 0.4 | -0.5 - 1.3 | 0.398 |
| Lymphocytes at admission (count/mm^3^) | 1508 |  |  |  |  |  |  |  |  |
| ≤4500 |  | 1491 (98.7%) | 4.5 (2.3 – 7.0) | 499 (33.5%) | 475 (31.9%) | 517 (34.7%) | 0 |  |  |
| >4500 |  | 17 (1.3%) | 3.0 (2.7 - 6.2) | 10 (58.8%) | 2 (11.8%) | 5 (29.4%) | -1.1 | -3.3 - 1.1 | 0.315 |
| Lymphocytes highest value (count/mm^3^) | 1508 |  |  |  |  |  |  |  |  |
| ≤4500 |  | 1494 (99.1%) | 4.5 (2.3 – 7.0) | 502 (33.6%) | 473 (31.7%) | 519 (34.7%) | 0 |  |  |
| >4500 |  | 14 (0.9%) | 3.1 (2.4 - 5.3) | 7 (50%) | 4 (28.6%) | 3 (21.4%) | -1.2 | -3.6 - 1.1 | 0.304 |
| Lymphocytes at discharge (count/mm^3^) | 1508 |  |  |  |  |  |  |  |  |
| ≤4500 |  | 1504 (99.8%) | 4.4 (2.3 – 7.0) | 508 (33.8%) | 474 (31.5%) | 522 (34.7%) | 0 |  |  |
| >4500 |  | 4 (0.2%) | 5.1 (4.4 - 5.2) | 1 (25%) | 3 (75%) | 0 (0%) | -1 | -5.4 - 3.4 | 0.657 |
| Hematocrit at admission (%) | 1508 |  |  |  |  |  |  |  |  |
| ≤47(F)/51(M) |  | 1441 (95.4%) | 4.4 (2.3 - 6.9) | 492 (34.1%) | 457 (31.7%) | 492 (34.1%) | 0 |  |  |
| >47(F)/51(M) |  | 67 (4.6%) | 5.4 (3.1 - 7.4) | 17 (25.4%) | 20 (29.9%) | 30 (44.8%) | 0.9 | -0.2 – 2.0 | 0.122 |
| Hematocrit highest value (%) | 1508 |  |  |  |  |  |  |  |  |
| ≤47(F)/51(M) |  | 1426 (94.4%) | 4.4 (2.3 - 6.9) | 489 (34.3%) | 453 (31.8%) | 484 (33.9%) | 0 |  |  |
| >47(F)/51(M) |  | 82 (5.6%) | 5.7 (3.2 - 7.4) | 20 (24.4%) | 24 (29.3%) | 38 (46.3%) | 0.8 | -0.2 - 1.8 | 0.114 |
| Hematocrit at discharge (%) | 1508 |  |  |  |  |  |  |  |  |
| ≤47(F)/51(M) |  | 1495 (99.1%) | 4.4 (2.3 – 7.0) | 506 (33.8%) | 472 (31.6%) | 517 (34.6%) | 0 |  |  |
| >47(F)/51(M) |  | 13 (0.9%) | 6.0 (3.2 - 8.2) | 3 (23.1%) | 5 (38.5%) | 5 (38.5%) | 3.7 | 1.2 - 6.1 | 0.003 |
| Urea at admission (mg/dl) | 1508 |  |  |  |  |  |  |  |  |
| ≤54 |  | 1289 (85.3%) | 4.3 (2.3 – 7.0) | 445 (34.5%) | 414 (32.1%) | 430 (33.4%) | 0 |  |  |
| >54 |  | 219 (14.7%) | 5.2 (2.5 - 8.1) | 64 (29.2%) | 63 (28.8%) | 92 (42%) | 0.6 | -0.1 - 1.3 | 0.052 |
| Urea highest value (mg/dl) | 1508 |  |  |  |  |  |  |  |  |
| ≤54 |  | 1211 (80.6%) | 4.3 (2.3 - 6.8) | 429 (35.4%) | 389 (32.1%) | 393 (32.5%) | 0 |  |  |
| >54 |  | 297 (19.4%) | 5.2 (2.8 - 8.1) | 80 (26.9%) | 88 (29.6%) | 129 (43.4%) | 0.8 | 0.2 - 1.4 | 0.006 |
| Urea at discharge (mg/dl) | 1508 |  |  |  |  |  |  |  |  |
| ≤54 |  | 1402 (92.8%) | 4.4 (2.3 - 6.9) | 481 (34.3%) | 443 (31.6%) | 478 (34.1%) | 0 |  |  |
| >54 |  | 106 (7.2%) | 4.9 (2.6 - 7.6) | 28 (26.4%) | 34 (32.1%) | 44 (41.5%) | 0.8 | -0.1 - 1.7 | 0.085 |
| CRP at admission (mg/dl) | 1508 |  |  |  |  |  |  |  |  |
| ≤5 |  | 337 (22.3%) | 3.8 (2.3 - 6.2) | 132 (39.2%) | 113 (33.5%) | 92 (27.3%) | 0 |  |  |
| >5 |  | 1171 (77.7%) | 4.6 (2.4 - 7.1) | 377 (32.2%) | 364 (31.1%) | 430 (36.7%) | 0.7 | 0.1 - 1.2 | 0.017 |
| CRP highest value (mg/dl) | 1508 |  |  |  |  |  |  |  |  |
| ≤5 |  | 109 (7.2%) | 3.6 (2.2 - 6.1) | 45 (41.3%) | 36 (33%) | 28 (25.7%) | 0 |  |  |
| >5 |  | 1399 (92.8%) | 4.5 (2.3 - 7.1) | 464 (33.2%) | 441 (31.5%) | 494 (35.3%) | 0.3 | -0.6 - 1.2 | 0.488 |
| CRP at discharge (mg/dl) | 1508 |  |  |  |  |  |  |  |  |
| ≤5 |  | 236 (15.6%) | 3.9 (2.3 - 6.5) | 91 (38.6%) | 77 (32.6%) | 68 (28.8%) | 0 |  |  |
| >5 |  | 1272 (84.4%) | 4.5 (2.4 - 7.1) | 418 (32.9%) | 400 (31.4%) | 454 (35.7%) | 0.2 | -0.5 - 0.8 | 0.624 |

Data presented as: n (%); median (25th percentile–75th percentile).

β: β coefficient; CI: Confidence Interval; AST: Aspartate Aminotransferase; ALT: [Alanine Aminotransferase](https://www.google.com/search?sxsrf=AJOqlzUK4IfUFGYstHQVXdErDugcDRNTNQ:1675800053858&q=alanine+aminotransferase&spell=1&sa=X&ved=2ahUKEwj21rLXmYT9AhVihv0HHSoUAkwQkeECKAB6BAgmEAE); GGT: Gamma Glutamyl Transferase; ALP: [Alkaline Phosphatase; F:female; M:male; CRP: C-Reactive protein.](https://www.medicalnewstoday.com/articles/321984#:~:text=The%20reference%20range%20for%20normal,30%E2%80%93120%20IU%2Fl.)

Supplementary Table 3. Number of patients and median waiting time by hospital.

| Hospital | N | Median waiting time (months) *^a^* |
| --- | --- | --- |
| General University Hospital Dr Balmis, Alicante | 171 | 3.6 (2.2 - 5.3) |
| University Hospital Josep Trueta, Girona | 40 | 4.0 (1.3 - 9.5) |
| University Hospital of Burgos, Burgos | 81 | 4.2 (2.2 - 7.1) |
| University Hospital of Donostia, Donostia | 77 | 4.7 (3.3 - 5.9) |
| University Hospital Gregorio Marañon, Madrid | 40 | 3.5 (2.5 - 5.9) |
| University Hospital La Princesa, Madrid | 38 | 2.7 (1.5 - 6.7) |
| University Hospital Rio Hortega, Valladolid | 89 | 4.1 (2.1 - 6.1) |
| University Hospital Ramon y Cajal, Madrid | 122 | 4.1 (2.2 – 7.0) |
| University Hospital San Jorge, Huesca | 71 | 5.6 (2.9 - 7.6) |
| University Hospital Santa Lucia, Cartagena | 95 | 2.3 (1.5 - 4.6) |
| University Hospital Son Espases, Palma de Mallorca | 130 | 5.6 (4.3 - 7.2) |
| University Hospital Arnau de Vilanova, Lleida | 82 | 3.9 (1.5 - 6.5) |
| University Hospital Galdakao, Vizcaya | 163 | 3.9 (2.5 - 6.8) |
| University Hospital Lozano Blesa, Zaragoza | 145 | 4.7 (2.6 - 7.5) |
| University Hospital Miguel Servet, Zaragoza | 138 | 7.2 (5.1 - 10.2) |
| University Hospital San Pedro, Logroño | 26 | 2.7 (2.0 - 4.6) |

Data presented as: n; median (25th percentile–75th percentile). *^a^p-value: <0.001*

# References

1. Banks PA, Bollen TL, Dervenis C, et al. Classification of acute pancreatitis 2012 ; revision of the Atlanta classification and definitions by international consensus. Gut. 2013; 62(1):102-11.

2. Kiriyama S, Kozaka K, Takada T, et al. Tokyo Guidelines 2018 ; diagnostic criteria and severity grading of acute cholangitis ( with videos ). J Hepatobiliary Pancreat Sci. 2018;25(1):17-30.

3. Yokoe M, Hata J, Takada T, et al. Tokyo Guidelines 2018 ; diagnostic criteria and severity grading of acute cholecystitis ( with videos ). J Hepatobiliary Pancreat Sci. 2018;25(1):41-54.

4. Wang DQH, Afdhal NH. Litiasis biliar. In; Feldman M, Friedman LS, Brandt LJ, eds. Sleisenger y Fordtran. Enfermedades Digestivas y Hepaticas. 10^a^. Barcelona. Elsevier, 2018;1100-1133.

5. Services H, Program DF, Affairs V, et al. Adapting a clinical comorbidity index for use with ICD-9-CM administrative databases. J Clin Epidemiol.1992; 45(6):613-9.
